# Efficacy for writing self-regulation, attitude toward writing, and quality of second grade students’ writing

**DOI:** 10.3389/fpsyg.2023.1265785

**Published:** 2023-10-17

**Authors:** Gustaf B. Skar, Steve Graham, Alan R. Huebner

**Affiliations:** ^1^Department of Teacher Education, Norwegian University of Science and Technology, Trondheim, Norway; ^2^MLF Teachers College, Arizona State University, Tempe, AZ, United States; ^3^Department of Applied and Computational Mathematics and Statistics, University of Notre Dame, Notre Dame, IN, United States

**Keywords:** writing, self-efficacy, attitudes, self-regulation, gender, language, text quality

## Abstract

Motivational beliefs, such as writing self-efficacy and attitude toward writing, are believed to foster or hinder writing by influencing if one chooses to write, how much effort is committed to writing, and what cognitive resources writers apply. In the current study, we examined self-efficacy for writing self-regulation and attitude toward writing of 2,124 Grade 2 Norwegian students (1,069 girls; 1,055 boys). We investigated if there were differences in each of these beliefs between girls and boys and students who differed in their language status (Norwegian first language, Norwegian and another language both first language, or language other than Norwegian first language). We further tested if each of these writing motivational beliefs made statistically unique contributions to predicting the quality of students’ writing. In each of these analyses, we controlled for variance related to individual- and school factors. Girls were more positive about writing than boys, and they were confident about their abilities to self-regulate writing. Students with Norwegian and another language both as first language (“bilingual” students) had a more positive attitude toward writing than the other two language groups. Efficacy for writing self-regulation and attitude toward writing both made statistically significant unique contributions to predicting the quality of students writing, although these two writing beliefs collectively accounted for just 2% of the variance in writing quality scores once individual- and school-factors were controlled. Recommendations for future research and implications of the finding are discussed.

## Introduction

During the last three decades, those who developed models and theories of writing placed increasing emphasis on the important role of motivational beliefs in writing. In his revision of the seminal Hayes and Flower (1980) model of writing, Hayes (1996) argued that “Motivation is manifest, not only in relatively short-term responses to immediate goals, but also in long-term predispositions to engage in certain kinds of activities” (p. 9). As a result, he revised the earlier model to indicate that writers’ motivational beliefs and attitudes influence and are influenced by the long-term memory and cognitive processes writers’ employ, and the interaction between cognitive and affective aspects of writing were essential to a full understanding of how writing operates.

In Zimmerman and Risemberg’s (1997) model of writing as a self-regulated process, writing was depicted as a complex cognitive task that is demanding, intentional, and self-sustaining, requiring a high-level of regulation on the part of the writer to manage covert writing processes, one’s writing behavior, and the writing environment. Writers exert control over internal personal factors, behavioral patterns, and environmental attributes by employing a variety of self-regulation strategies (e.g., goal setting, planning, seeking information, evaluating). As these strategies are employed, writers monitor and react to self-feedback or feedback from others to determine which strategies were or were not successful. This feedback influences which self-regulation strategies are applied in the future. It also influences a writer’s sense of efficacy, as beliefs about writing competence are presumably strengthened or weakened depending upon the perceived success of the deployed self-regulation strategies. In turn, self-efficacy beliefs are believed to influence motivation to write, use of self-regulatory strategies, and one’s success when writing.

The more recent writer(s)-within-community model (WWC; Graham, 2018), also assigned a central role to writing motivational beliefs. A basic premise of this model was that motivation beliefs foster or hinder writing, influencing whether one writes, how much effort is committed, what cognitive resources and processes writers apply; which tools are used to create writing; how one monitors and regulates the composing process; and how one interacts with others while writing or learning to write. Accordingly, writers employ a variety of motivational beliefs about writing which interact to influence what a writer does. This includes beliefs about writing efficacy, attitudes toward writing, value and utility of writing, motives for writing, reasons for writing success or failure, goal orientation for writing, and writing identity. We provide two examples of how such beliefs can interact, using self-efficacy for writing and attitude toward writing to illustrate this principle. Students who are confident about their writing competence may develop a positive attitude about writing because their perceived efficacy leads them to view writing in an optimistic light, resulting in commitment and effort when writing. In contrast, students may have a neutral or negative attitude toward writing, but still evidence considerable commitment and effort when writing, because they are confident about their capabilities to complete writing tasks successfully.

To date, the two writing motivational beliefs that have received the most attention in the research literature are self-efficacy and attitude toward writing. In a systematic review of 84 writing motivational studies, Camacho et al. (2020) indicated that 44 and 27% of the studies reviewed included measures of efficacy and attitudes, respectively. While some studies have examined the writing attitudes of beginning writers (grades two and below; e.g., Graham et al., 2007, 2012; Skar et al., 2022, 2023), including the relationship between attitudes and writing performance (Knudson, 1992; Olinghouse and Graham, 2009), fewer studies have examined the self-efficacy of such young writers (e.g., Guay et al., 2016; Schrodt et al., 2019; Traga Philippakos and MacArthur, 2020). We were unable to locate any investigations that assessed relations between writing efficacy and writing performance with such young children.

The current study addressed the relative lack of knowledge about writing efficacy and attitude with beginning writers in four important ways. One, we examined the writing attitude and efficacy of a large sample of second grade students in Norway (*N* = 2,842). This is the first study to our knowledge to examine both of these writing beliefs concurrently with such young writers in Norway or in any other country. Two, we examined if student-level factors [gender, age, and language status (Norwegian as first language, Norwegian and another language both as first language, or Norwegian as second language)] as well as school-level factors (school size, national test scores, proportion of certified teachers, school hours per student, and students per special education teacher) were related to each of these motivational constructs. Such an analysis between writing motivational variables and multiple student- and school-factors has not previously been conducted. While previous studies examined relations between these motivational beliefs and gender and age (Ekholm et al., 2018; Camacho et al., 2020) as well as language status (Busse et al., 2023), no study has yet examined the collective contribution of these individual- and school-level factors to predicting self-efficacy for writing or attitude toward writing.

Three, we examined if writing efficacy and attitudes each made a unique contribution to predicting writing quality, after controlling for variance associated with the individual- and school-level factors describe above as well as students’ scores on a handwriting fluency measure and the other motivational belief. This is the first time that an analysis where variance due to such an array of individual- and school-level variables were first controlled has been undertaken. Finally, we focused our examination of writing efficacy on second-grade students perceived competence to self-regulate their writing. No previous study has examined this aspect of writing self-efficacy with students this young. Zumbrunn et al. (2020) did examine if self-efficacy for writing self-regulation predicted the writing of students as early as Grade 3. However, they assessed writing performance using teacher grades for writing (we assessed students’ actual writing products). Further, their analyses did not examine if self-efficacy for writing self-regulation predicted writing performance with Grade 3 students specifically. Rather, they evaluated this association between students in Grades 3 to 10 collectively. Across this broad range of students, they did not find a statistically significant relation between self-efficacy for writing self-regulation and the writing grades assigned by teachers.

It is especially important to learn more about beginning writers’ efficacy for writing and their attitudes toward writing because it is possible that motivational beliefs formed early have long-lasting effects (Bandura, 1986). Students typically start school with a positive attitude about writing and a belief they can write (Calkins, 1983; Graham et al., 2007), but some studies show that developing writers become less positive and efficacious about writing over time (Knudson, 1991; Pajares, 2003). As a result, it is imperative that we document beginning writers’ efficacy and attitudes toward writing as well as identify individual- and school-level factors that predict these beliefs. It is further important to determine if young children’s attitudes and efficacy beliefs predict how well they write because it is not clear at this point when this occurs.

Before presenting our research questions and hypotheses, we first examine the constructs of self-efficacy for writing and attitude toward writing as operationalized in this investigation. At the same time, we review prior research with older children examining if these two constructs predict students’ writing.

### Self-efficacy for writing self-regulation

Because writing is a complex task requiring the management and orchestration of writing skills, processes, knowledge, beliefs, and behaviors as well as the governance of the environment where writing takes place (Hayes, 1996; Zimmerman and Risemberg, 1997; Graham, 2018), self-regulation is essential to effective writing. Beginning writers commonly apply an approach to writing that minimizes some self-regulatory activity by converting the writing process into telling what one knows, with little attention directed at whole text organization, needs of the reader, or constraints imposed by the writing topic (e.g., Scardamalia and Bereiter, 1985). Nevertheless, these children must still identify the purpose for their writing, initiate and sustain the writing process, avoid distractions while writing, and continue to write even when it is difficult (Bruning et al., 2013). Writing cannot be accomplished if these self-regulatory skills are not applied and, as Pajares (2003) indicated, developing writers’ efficacy for employing these skills influences their use and students’ writing success.

To measure self-efficacy for writing self-regulation, we asked participating students in this study to complete the self-efficacy for writing self-regulation scale designed and tested by Bruning et al. (2013). This scale assesses students’ perceived capabilities to manage the writing task (start and sustain writing), avoid disruptions and control frustrations while writing, and set writing goals (the same basic self-regulatory strategies described above). A number of studies have demonstrated that scores on this measure predict one or more aspects of upper-elementary and secondary students’ writing. In a study with Grade 5 students in the US, Wijekumar et al. (2019) found that self-efficacy for writing self-regulation predicted the length and quality of students’ writing. De Smedt et al. (2016) reported that this measure predicted Grade 5 and 6 Flemish students’ reported use of self-regulation when writing. In a study conducted in Portugal, Limpo and Alves (2017) found this measure predicted the overall quality of essays produced by Grade 7 and 8 students. Further, Bruning et al. (2013) indicated self-efficacy for writing self-regulation predicted US high school students self-reported writing capabilities as well as their scores on a state wide writing assessment. Even so, this particular measure of self-efficacy was not statistically related to the writing of Grade 5 students or high school students in two studies conducted in the US (Yilmaz Soylu et al., 2017; Graham et al., 2019).

As noted earlier, there is currently no data on how beginning writers in second grade or below view their efficacy for writing self-regulation or if these views are related to their gender, primary language, or the quality of their text. It is important to examine such relationships with these young writers though. Berninger and Amtmann (2003) indicated that beginning writers, such as the second-grade students in this study, are “dependent on other-regulation in the form of guided assistance from parents, teachers, and peers” (p. 350). If this is the case, then there is likely to be little to no relationship between self-regulation and writing for these students, and by extension little to no relationship between efficacy for writing self-regulation and students’ writing. The present study provides information that bears directly on Berninger and Amtmann’s (2003) claim.

### Attitude toward writing

Researchers have been inconsistent in how they define writing attitudes, even though the study of attitudes has played a prominent role in psychological research over time (Allport, 1954). According to Ekholm et al. (2018), attitudes can be defined as a generic or domain-specific disposition. Attitudes can also be viewed from a state or trait perspective (Camping et al., 2020). One can have a positive or negative disposition regarding a specific task (state) or a positive or negative disposition toward such tasks in general.

Ekholm et al. (2018) also indicated attitudes are characterized by affective and cognitive components. This was evident in the attitude toward writing measure applied in the current study. Students were asked to indicate their agreement with items assessing enjoyment to write (affective) and satisfaction with effort expended when writing and the resulting written product (cognitive).

All but one of the items used to assess writing attitudes (e.g., enjoyment of writing in general) were directly linked to the writing tasks students completed in this study. We felt that asking students about their attitude toward a specific task would make the task more concrete and understandable for the young children participating in this study, increasing the probability of obtaining a more valid test of the link between attitude toward writing and the writing students did in this study. In summary, the attitude toward writing measure in this investigation can be characterized as a disposition to respond favorably or unfavorably to a recent writing task as well as positive or negative judgments about effort expended and the resulting written product.

In their review of the research literature on students’ attitude toward writing, Ekholm et al. (2018) noted relatively few studies examined the relationship between writing attitudes and students’ writing performance. Of the studies that did examine this relationship, most found a positive relationship between attitudes and writing outcomes (Graham et al., 2007, 2012, 2017; Lee, 2013). With the exception of one study with middle school students, these investigations all involved students in the elementary grades, including students as young as 6 years of age (Graham et al., 2017). While attitude toward writing has typically predicted how well students write (see also Graham et al., 2019), this has not been the case in several investigations (e.g., Olinghouse and Graham, 2009; Wijekumar et al., 2019).

### Research questions and predictions

The present study was designed to answer the following questions:

1. Is self-efficacy for writing self-regulation related to Grade 2 students’ gender and language status after controlling for individual- and school-level factors? (RQ1)
2. Is attitude toward writing related to Grade 2 students’ gender and language status after controlling for individual- and school-level factors? (RQ2)
3. Do self-efficacy for writing self-regulation and attitude toward writing each make unique statistical contributions to predicting the quality of Grade 2 students’ writing after controlling for individual- and school-level factors as well as the other motivational belief? (RQ3)

For all three research questions, the school-level control variables were school size, national test scores, proportion of certified teachers, school hours per student, and number of students per special education teacher. We controlled for variance related to these factors because previous research demonstrated school-level factors predict students’ writing performance (e.g., Walberg and Ethington, 1991), and theoretically students’ writing beliefs and performance are shaped and constrained by the communities in which they write and learn to write (Graham, 2018).

Students’ age was also treated as a control variable for each research question. While the contribution of this variable to predicting writing beliefs and performance is likely to be minimal because all students were in Grade 2, we felt it was still important to control for variance related to age because readiness factors and experience writing likely play a role in young students’ development of writing beliefs and writing performance. When examining if gender or language status predicted self-efficacy for writing self-regulation (RQ1), we also treated attitudes toward writing as a control variable. Likewise, when determining if gender or language status predicted attitude toward writing (RQ2), self-efficacy for writing self-regulation was treated as a control variable. We did this because scores on self-efficacy and writing attitude measures are statistically related to each other (Pajares, 2003; Ekholm et al., 2018; Camacho et al., 2020).

For RQ3 which examined the predictive value of self-efficacy for writing self-regulation and attitude toward writing, we further controlled for students’ gender, language status, and handwriting fluency. Gender and language status were statistically related to writing beliefs and writing quality in previous studies (e.g., Reilly et al., 2018; Camping et al., 2020). This was also the case for handwriting fluency (Graham et al., 1997; Kent and Wanzek, 2016; Skar et al., 2022). By controlling for variance related to these and the other variables described above, we added greater precision to all of our analyses because these variables can potentially confound the primary relationships we were investigating.

We hypothesized gender and language status would each make a unique and statistically significant contribution to predicting self-efficacy for writing self-regulation and attitude toward writing (RQ1 and RQ2). As Pajares (2003) concluded in his review of writing self-efficacy research, girls are more efficacious about their writing competence than boys. Likewise, in their review of research on writing attitudes, Ekholm et al. (2018) reported that girls have more positive attitudes than boys (Ekholm et al., 2018). While there is no current systematic review of relations between writing motivational beliefs and students’ language status, individual studies such as those conducted by Camping et al. (2020, 2023) demonstrated that language status predicts students’ beliefs about writing.

We further hypothesized that self-efficacy for writing self-regulation and attitude toward writing would each make a unique and statistically significant contribution to predicting the quality of Grade 2 students’ writing (RQ3). Klassen (2002) and Pajares (2003) in their reviews of the writing self-efficacy literature reported that self-efficacy for writing consistently predicts writing performance, and this is evident in studies with older students by Bruning et al. (2013), De Smedt et al. (2016), Limpo and Alves (2017), and Wijekumar et al. (2019). Similarly, in their review of the literature, Ekholm et al. (2018) found that variation in writing performance was predicted by students’ attitudes toward writing, as evidenced in studies by Graham et al. (2007), Graham et al. (2012, 2017, 2019), and Lee (2013). However, given Berninger and Amtmann’s (2003) contention that beginning writers are dependent on other forms of regulation (e.g., guided assistance from parents, teachers, and peers), it is possible that self-efficacy for writing self-regulation will account for just a small amount of the variability in students’ writing scores.

## Materials and methods

### Context of the study

This investigation was conducted in Norway, in which writing has been a “key competency” since an educational reform in 2006 (Skar and Aasen, 2021). In the Norwegian setting, “key competency” refers to skills and competencies that should be taught across the curriculum. The other key competencies are English, ICT skills, mathematics, and reading. Although writing is posited as a fundamental skill, previous research (Håland et al., 2019; Graham et al., 2021) have found indications of great variation in terms of time devoted to writing instruction and in terms of contents of writing instruction among elementary school teachers in Norway. The status of writing in Norwegian schools are also blurred by the fact that there are no explicit learning objectives tied to any of the key competencies in the obligatory national curriculum. However, there are national tests in English, mathematics, and reading providing indirect attainment goals in terms of proficiency levels and national norms. Such tests are not available for writing. Previous research has indicated that 17% of students in first grade may struggle to develop appropriate writing skills (Skar and Huebner, 2022), but these estimates stem from analysis of a small sample (*N* = 832) of students.

### Participants

Participants were 2,124 Norwegian second grade students who completed all measures administered in this investigation. The sample represented 74.7% of students from a larger sample of students (*N* = 2,842), whose parents gave permission for their children to participate. The participants represented 143 classrooms in 57 public schools, and 3.5% of all second-grade students in Norway 2021. Students in the current sample attended schools that were involved in a writing instructional study in the academic years of 2019–2021, and data for this investigation was collected in May and June of 2021 (i.e., at the end of that study). There were 1,055 boys (49.7%) and 1,069 girls (50.3%) in the sample, 1,710 students (80.5%) who had Norwegian as their first language, and 246 students (11.6%) who had Norwegian and another language as their first languages (“Bilingual”). One hundred fifty-eight students (7.4%) had another language than Norwegian as their first language (“Other”). We had no information about language for ten participants (0.5%). Please refer to Table 1 for a sample breakdown by gender and language status.

**TABLE 1:** Means and standard deviations for handwriting fluency, text quality, attitude, and self-efficacy by gender and language status.

			HWF		TQ		Attitude		Self-efficacy	
**Gender**	**Language**	***N***	**Mean**	***SD***	**Mean**	***SD***	**Mean**	***SD***	**Mean**	***SD***
Boys	Bilingual	127	26.3	11.6	3.1	0.6	2.4	0.5	4.4	1.3
	Norw	847	22.5	9.9	3.1	0.5	2.3	0.5	4.3	1.2
	Non-Norw	78	25.2	11.9	3.1	0.6	2.6	0.5	4.5	1.2
Girls	Bilingual	119	26.8	11.9	3.3	0.5	2.6	0.4	4.8	1.0
	Norw	863	29.1	11.6	3.4	0.5	2.6	0.4	4.7	1.0
	Non-Norw	80	25.4	13.6	3.0	0.5	2.6	0.4	4.5	1.2

HWF, handwriting fluency; TQ, text quality; Bilingual, Norwegian and another first language; Norw, native Norwegian speaker; Non-Norw, non-native Norwegian speaker.

We believe the sample in our investigation was representative of second graders in Norway based on the following comparisons between sample and population characteristics. First, the proportion of boys in our sample (49.7%) was similar to that of the population (51%).^1^ Second, our sample included 7.4% of students with another language than Norwegian as their mother tongue, which was similar to the national proportion (8.7%) of Norwegian second graders who in 2021 were entitled to mother tongue education for students with another language than Norwegian. Third, students in our sample were drawn from five municipalities, which reflected the diverse sizes of municipalities in Norway. These municipalities ranged from large (*N* = 709,037; 12.9% of Norway’s population; 46 times larger than the average municipality) to medium-sized (*N* = 14,623; 2.6% of Norway’s population; 95% the size of an average municipality) to small (*N* = 2,431; 0.04% of Norway’s population; 15.8% the size of an average municipality). Fourth, our sample included municipalities from various regions of Norway, encompassing both urban and rural areas.^2^ Fifth, the proportion of certified teachers in the schools from which students were drawn (*M* = 95.8%, *SD* = 5.4%) closely aligned with the percentage of certified teachers in all schools across Norway (*M* = 95%). Sixth, there were 84.8 students per special education teacher in our sample (*SD* = 33.1), while the national average was 82.4 (*SD* = 98.2).

It should also be noted that the average number of “school hours” per student (i.e., instructional hours divided by the number of students) in our sample was 54.5 (*SD* = 12.4), slightly lower than the national average of 61 h. Schools in our sample were larger in terms of student population (*M* = 482.5, *SD* = 174.3) when compared to the average schools in Norway (*M* = 225, *SD* = 166). Further, schools in our sample had similar, albeit slightly higher average score on the 5th grade national tests in reading, mathematics and English^3^ (*M* = 51.5, *SD* = 2.82, score range: 45.8 to 56.6), than schools in Norway in general (*M* = 50, *SD* = 10, score range: 36 to 68), according to data from the Directorate for Education and Training.

### Measures

#### Self-efficacy for writing self-regulation

Self-efficacy for writing self-regulation was measured using an already established self-efficacy scale (Bruning et al., 2013). The scale consists of six statements: (1) I can focus on my writing for at least 1 h; (2) I can avoid distractions while I write; (3) I can start writing assignments quickly; (4) I can control my frustration when I write; (5) I can think of my writing goals before I write; (6) I can keep writing even when it’s difficult. For this investigation, the statements were translated into Norwegian. Participants in the validation study for this scale (Bruning et al., 2013) were asked to indicate their agreement on a scale that ranged from 0 to 100 (i.e., effectively 0–100%). Given the age of the students in this investigation, we opted for a shorter range, expressed in a more familiar way. In Norway, it is customary to express appraisals using dice. For instance, a movie may be awarded five dice, while a book may receive three dice. Consequently, we asked students to indicate their agreement with each item using a die with one dot (lowest agreement) to dice with six dots (highest agreement). To derive a score for this measure, we averaged a student’s score across the six items. A higher number indicated greater self-efficacy for regulating writing. Reliability for the measure was acceptable (Cronbach’s alpha = 0.78), although somewhat smaller than the one reported by Bruning et al. (2013) (Cronbach’s alpha = 0.88). A confirmatory factor analysis with a one factor solution showed acceptable fit (standardized root mean square residual (SMRM) = 0.016; root mean square error of approximation (RMSEA) = 0.034; comparative fit index (CFI) = 0.992; Tucker Lewis index (TLI) = 0.986), albeit the chi-square statistic was significant (which is often the case in large sample studies; Brown, 2015).

#### Attitude toward writing

Attitude toward writing was assessed with a four-item scale, which was validated with students in Grades 1 to 3 in a previous investigation (Skar et al., 2022). The scale contained items which asked students to indicate: (1) how much they enjoyed their most-recent writing task, (2) how satisfied they were with their most-recent text they created, (3) how satisfied they were with their effort during their most-recent writing task, and (4) how much they enjoyed writing in general. Students were asked to indicate their answers using a three-point scale (designed as stars), with a higher number of stars indicating more enjoyment and satisfaction, respectively. The students took the attitude toward writing scale twice, once after each time they wrote a text. To derive a score for this measure, we averaged a student’s score across all four items. The scale reliability was acceptable (Cronbach’s alpha = 0.81), and somewhat higher than estimated in the validation study higher (Cronbach’s alpha = 0.71; Skar et al., 2022).

#### Text quality

Text quality was measured by having students complete two “purposeful writing” tasks. The tasks, which were designed within the context of a Norwegian writing intervention program (Skar et al., 2023), asked students to describe to researchers what they enjoyed doing in recess time, and what happened on a day where they found a magical hat. In both instances, the students’ teacher introduced the topic and conducted a brainstorming session focusing on the communicative purpose (i.e., to describe to someone external to the school context, and write a fictitious recount for entertainment purposes) and possible content. The teacher based the discussion about content on a picture supplied by the researcher. For the recess time task, a picture of young students in a playground was shown, and for the magical hat task, a picture of a hat with stars above it laying on a gravel road was shown. When the teacher deemed that students were sure about why to write and what to write, the writing commenced, and students would—in keeping with standard Norwegian procedures—be granted 45 min to complete each task. The distribution of tasks was counterbalanced so that half of the students wrote about recess time first, and the other part about the magical hat.

### Scoring

Students’ texts were rated by 24 trained raters. Each text was rated by two independent raters, and raters marked an average of 488.5 texts (*SD* = 107.5) per person. There were 50 anchor texts, which had been used in previous rating sessions in the context of the intervention study, which served as “linking devises,” so that all raters could be linked to all other raters.

The raters used an assessment rubric with eight five-point text quality rating scales which had been validated previously (Skar et al., 2020a,b). The eight rating scales tapped into different aspects of text quality, and common for all scales was that a higher number indicated higher quality. They were: audience awareness, organization, content relevance, vocabulary, sentence construction, spelling, legibility and punctuation. Audience awareness focused on textual indications that the writer was concerned about his/her reader (e.g., by adding a greeting phrase, or by explaining an uncommon concept). Organization concerned the macro and micro structure of the text. Content relevance concerned the proportion of the text that contained information relevant vis-a-vis the writing task (e.g., text about recess activities rather than text about other aspects of a person’s life). Vocabulary concerned the repertoire of words in the text, and sentence construction, spelling legibility and punctuation tapped into texts’ sentence construction (including grammar), spelling, legibility and punctuation (the criteria can be found in an online Supplementary Appendix).

We derived text quality scores for students by fitting the data to the many-facet Rasch measurement (MFRM) model (Linacre, 1994; Eckes, 2011). The following Rasch model was used:

$$l\,o\,g\,\left(\frac{P_{i\,j\,m\,k\,l}}{P_{n\,i\,j\,m\,k\,\left(l-1\right)}}\right)=\beta_{i}-\tau_{j}-\delta_{m}-\gamma_{k}-\varphi_{l}$$

where *P*_*ijmk(l)*_ represents the probability of student *i*, on task *j*, rating scale *m*, by rater *k*, receiving a score of *l*, and *P*_*ijmk(l–1)*_ represents the probability of the same student under the same conditions receiving a score of *l*-1. β_*i*_ is the ability for person *i*, τ_*j*_ the difficulty of task j, δ_*m*_ is the difficulty of rating scale *m*, and γ_*k*_ is the severity of rater *k*. Finally, φ_*l*_ represents the point on the logit scale where category *l* and *l*–1 are equally probable.

The analysis yielded a single text quality score per student. This was the “fair average” output from the FACETS (Linacre, 2017) software, which was the average score across tasks, rating scales and raters while controlling for variations in difficulty and severity. Fair scores ranged from 1 to 5 and were not restricted to integers. The data fitted the MFRM model well. First, the reliability of separation (analogous to Cronbach’s alpha) was *R* = 0.95, indicating that we were able to separate student proficiency with a high precision.^4^ Second, there were 4.5% standardized residuals exceeding |3| and 0.5% residuals exceeding |2|, which was within the boundaries of what is generally excepted as good fit (Eckes, 2011).

#### Handwriting fluency

Teachers administrated a copying task designed to assess students’ handwriting fluency. The task, which has been used in the US (Graham et al., 1997) and in Norway (Skar et al., 2022) with similar samples of students. The paragraph students are asked to copy was taken from Group Diagnostic Reading and Aptitude and Achievement Tests (Monroe and Sherman, 1996). Students were presented with a paragraph and were provided with 90 seconds copy the paragraph as quickly and correctly as they can. To assist students in completing this task correctly, students were shown a video that reviewed the steps for completing the task. Teachers were asked to show the video more than once if students did not appear to comprehend what they were to do. The teachers were further asked to start the test when all students sat with pencils in hand and paragraph in front of them. When starting the test, the teacher started a timer provided by researchers. The teachers were instructed to instruct students to stop copying the paragraph when the timer alarm rang.

To derive a measure of handwriting fluency, the number of correct letters copied were tallied and divided by 1.5, which yielded a measure of letters copied correctly per minute. Tallying was done by personnel who had vast experience of coding similar tasks at the first author’s university. Ten per cent of student text samples were double coded to estimate reliability, which was acceptable (κ = 0.812, ICC = 0.99).

#### Language background

Information about students’ language background was obtained from students’ teachers. Teachers indicated whether Norwegian was a student’s first language or second language or whether a student was bilingual, with Norwegian and another language both as native languages.

### Procedures

Data was collected within the context of a large-scale writing research project,^5^ and all data collection was performed by students’ teachers. We opted for teacher-led data collection for two reasons. First, it is uncommon for young students to participate in standardized testing activities. Formal grades are not introduced until Grade 8, and students sit for their first high stake test in Grade 10 in Norway. We suspected that letting teachers administrate the tests in the frames of ordinary instruction would lower the risk of students feeling uncomfortable or pressured by the testing situation. Second, the scale of the project made it impossible for us to administrate all tests.

To reduce possible variations of how measures were administrated, we gave teachers detailed instructions on how to administer the tests. We supplied teachers with written instruction for each test (two “purposeful writing” tasks, one copy task, two attitude tasks, and one self-efficacy task). We also supplied teachers with video instructions on how to perform the “purposeful writing” tasks and the copying task. Students were also shown the video for the copying task. Further, all teachers were invited to online seminars in which the research team provided information on how tasks should be administrated. In these latter seminars we stressed that teachers should only proceed with test administration after they had assessed their students to understand the task at hand.

Test administration took place in a fifteen-day window in May and June of 2021, and task administration was counterbalanced. Once teachers had completed the administration of all tasks, student responses were sent by mail to the research team. All texts were anonymized and information about gender and language background was masked prior to coding.

#### Statistical analysis

Before conducting statistical analyses, the two scores for writing quality were averaged together to obtain a single score used in all analyses. The same procedure was applied with the two attitudes toward writing scores.

Various statistical models were fit to examine the effects of several independent variables on three dependent variables: (1) scores for self-efficacy for writing self-regulation; (2) scores for attitude toward writing; and (3) text quality scores. Since students were nested within classrooms which were nested within schools, linear multilevel regression models (MLMs) were used to account for this clustered structure of the data. Specifically, the cluster structure resulted in the use of linear models with three levels, where students, classrooms, and schools were denoted as levels 1, 2, and 3, respectively. For both dependent variables, a “null” model with no predictors was fit to assess the correlation structure of the data resulting from the clustering. This correlation was expressed using intraclass correlation coefficients (ICCs). For three-level MLMs, there are two possible ICCs, one expressing the correlation between two students randomly sampled from the same class (same school) and one expressing the correlation between two students randomly sampled from the same school (different classes).

Next, for each dependent variable, models were fit containing both student-level (level 1) predictors and school-level (level 3) predictors. For the model with self-efficacy for writing self-regulation as the dependent variable, the student-level predictors included gender, language, and age (a control variable expressed in months). The school-level control variables included school size, national test scores, proportion of certified teachers, school hours per student and students per special education teacher. The model with attitude toward writing as the dependent variable applied the same student- and school-level predictors and control variables. The model with text quality as the dependent variable had the same student- and school-level predictors and control variables described for the first model, as well as handwriting fluency mean, attitude toward writing mean, and self-efficacy for writing self-regulation as level-1 predictors. In the models for all three dependent variables, the gender predictor was binary, and the language predictor had three levels: (1) native Norwegian speaker, (2) bilingual, and (3) a language other than Norwegian as the primary language. The native Norwegian level of the language predictor was taken as the reference level, and this contributed to the model coefficients for the other two levels. All other predictors were numeric.

The numeric predictors, as well as the binary gender predictor, were centered according to the recommendations of Enders and Tofighi (2007) and Brincks et al. (2017). Specifically, the student level predictors were centered relative to the mean of classroom to which the student belonged. Enders and Tofighi (2007) state that this centering within cluster approach, as opposed to centering relative to the grand mean, results in a pure estimate of the student-level relation between the predictor and dependent variable. On the other hand, the school-level predictors were centered according to the grand mean, as that is the only option for the highest level of the hierarchy.

## Results

### ICCs

The estimated variance components and ICCs obtained from the null models for both dependent variables are displayed in Table 2. The correlation due to clustering was stronger for text quality than for self-efficacy for writing self-regulation and attitude for writing. Specifically, the estimated correlation of text quality for two randomly selected students in the same classroom (same school) was 0.211 versus 0.059 for self-regulation and 0.076 for attitude. Similarly, the estimated correlation of text quality for two students in the same school (different classroom) was 0.122, versus 0.015 for self-efficacy for writing self-regulation and 0.040 for attitude.

**TABLE 2:** Variance components and ICCs for dependent variables.

Quantity	Self-efficacy	Attitude	Text quality
$\sigma_{e}^{2}$ (student variance)	1.246	0.196	0.238
$\sigma_{c}^{2}$ (class variance)	0.058	0.008	0.027
$\sigma_{s}^{2}$ (school variance)	0.020	0.008	0.037
ICC (class)	0.059	0.076	0.211
ICC (school)	0.015	0.040	0.122

Attitude, attitude toward writing.

### Self-efficacy as the dependent variable

Table 3 displays regression results for the linear MLM with self-efficacy for writing self-regulation as the dependent variable. The only predictor that was statistically significant was gender: on average, girls score about 0.341 points higher, on average, than boys. The model *R*^2^ was computed using the method described by Snijders and Bosker (2012) for three-level MLMs. The estimated value of *R*^2^ was 0.050, indicating that the predictors explained 5% of the variation in self-regulation scores.

**TABLE 3:** Regression results for linear MLM with self-efficacy as the dependent variable.

Para-meter	Estimate	Std. error	*t*-value	*P*-value
Intercept	4.562	0.044	102.866	<0.001
Gender: girls	0.341	0.051	6.708	<0.001
Language: bilingual	0.010	0.084	0.121	0.903
Language: other	−0.007	0.105	−0.070	0.944
Age	0.004	0.008	0.591	0.555
School size	0.000	0.000	0.784	0.437
Nation test	0.023	0.017	1.372	0.178
Proportion cert	−0.009	0.010	−0.919	0.364
Students/Sp. Ed	0.002	0.001	1.448	0.155
Hours	0.003	0.005	0.523	0.604

Model *R*^2^ = 0.050.

### Attitude toward writing as the dependent variable

Table 4 provides the regression results for the model with attitude as the dependent variable. The results are similar to the model with self-efficacy as the dependent variable, as gender was a statistically significant predictor in both models, the school-level variables were not significant in either model, and both models showed a relatively weak *R*^2^ value (0.092 for the model predicting attitude). Interestingly, however, language turned out to be a statistically significant predictor of attitude but not self-efficacy. As seen in Table 4, on average, bilingual students scored 0.076 points higher on attitude than students with Norwegian as their first language, and students that had another language than Norwegian as their first language scored about 0.119 points higher on attitude than students with Norwegian as their first language.

**TABLE 4:** Regression results for linear MLM with attitude as the dependent variable.

Para-meter	Estimate	Std. error	*t*-value	*P*-value
Intercept	2.448	0.020	123.334	< 0.001
Gender: girls	0.241	0.020	12.279	< 0.001
Language: bilingual	0.076	0.033	2.311	0.021
Language: other	0.119	0.042	2.867	0.004
Age	−0.003	0.003	−0.968	0.333
School size	0.000	0.000	0.893	0.377
Nation test	0.001	0.008	0.084	0.933
Proportion cert	0.007	0.004	1.670	0.103
Students/Sp. Ed	0.000	0.001	0.647	0.521
Hours	0.003	0.002	1.336	0.189

Model *R*^2^ = 0.092.

#### Text quality as the dependent variable

Table 5 presents the regression results for the MLM with text quality as the dependent variable. Similar to the model above, gender was statistically significant: girls scored about 0.165 points higher than boys, on average. In addition, handwriting fluency, attitude, toward writing, self-efficacy for writing self-regulation, and language status were statistically significant level-one variables, and national test scores was a statistically significant level-three variable. For example, for every one-unit increase in attitude toward writing, we expect text quality to increase by 0.121. Also, the model *R*^2^ was 0.311, signifying that the predictors and control variables collectively explained a bit over 30% of the variation in text quality.

**TABLE 5:** Regression results for linear MLM text quality as the dependent variable.

Para-meter	Estimate	Std. error	*t*-value	*P*-value
Intercept	3.235	0.029	110.586	<0.000
HWF	0.018	0.001	19.443	<0.000
Attitude	0.121	0.024	5.015	<0.000
Self-efficacy	0.046	0.009	4.947	<0.000
Gender: girls	0.165	0.020	8.389	<0.000
Language: bilingual	−0.015	0.032	−0.476	0.634
Language: other	−0.176	0.042	−4.208	<0.000
Age	0.009	0.003	3.349	0.001
School size	0.000	0.000	0.146	0.884
Nation test	0.043	0.012	3.683	0.001
Proportion cert	0.007	0.007	1.002	0.322
Students/Sp. Ed	0.000	0.001	0.017	0.987
Hours	0.001	0.003	0.234	0.816

Model *R*^2^ = 0.311.

While Table 5 displays the statistical significance of the predictors, it is also useful to assess their practical significance, i.e., their ability to explain the variation in the dependent variable text quality. To this end, Table 6 displays the variance in text quality explained (i.e., the amount of *R*^2^ contributed) for self-efficacy, attitude toward writing, and self-efficacy and attitude toward writing taken together. For example, for the first row in Table 6, the model was refit with the self-efficacy of self-regulation for writing removed. This model had a *R*^2^ of 0.305. Comparing to the full model with *R*^2^=0.311, we conclude that the self-efficacy predictor contributed approximately 0.311–0.305 – 0.006 to the *R*^2^ of the full model. Thus, while Table 5 shows that self-regulation had a statistically significance (i.e., a “real”) effect, Table 6 shows that this effect was weak. Coincidentally, attitude toward writing contributed approximately the same amount of *R*^2^ of the full model, so our conclusions about attitude toward writing were essentially the same as for self-efficacy of self-regulation for writing.

**TABLE 6:** Contributed R^2^ for self-efficacy, attitude toward writing, and both collectively.

Variable removed	*R* ^2^	Difference from full model (i.e.,*R*^2^ contributed by variables)
Self-efficacy	0.305	0.006
Attitude	0.305	0.006
Self-efficacy + attitude	0.290	0.021

## Discussion

In the current study, we examined if gender and language status of Grade 2 Norwegian students each made a separate and unique contribution to predicting the writing motivational beliefs of self-efficacy for writing self-regulation and attitude toward writing. Even more importantly, we examined if these two writing motivational beliefs each made separate contributions to predicting the quality of students’ writing. To enhance the precision of our analyses, we controlled for variance related to the contribution of multiple individual- and school-level factors.

### Efficacy, attitudes, gender, and language status

The Grade 2 Norwegian students in this study were confident in their capabilities to self-regulate their writing and they expressed a highly positive attitude toward writing. On a 6-point scale, with a score of 6 representing the highest level of confidence, the average score of participating Grade 2 students was 4.50. Young students have evidenced high-levels of efficacy in other studies (e.g., Pajares and Schunk, 2001: Traga Philippakos and MacArthur, 2020; Traga Philippakos and Voggt, 2021). There are multiple possible reasons for this including difficulty assessing efficacy at such a young age or over-estimating efficacy as a protective mechanism (e.g., to hide that writing can be difficult). In any event, research is needed to replicate our finding with such young children and better explore why efficacy is so elevated if our finding is replicated.

Likewise, on a 3-point scale, with a score of 3 representing the most positive attitude toward writing, students’ average score was 2.50. It should be noted that variability was particularly pronounced for the self-efficacy scores for writing self-regulation, with the standard deviation exceeding 1 point of the 5-point scale. It was slightly less pronounced for attitude toward writing, with a standard deviation of about one-half of a point on the 3-point scale. Consequently, many students’ scores on the self-efficacy and the attitude toward writing scales were very close to the ceiling score for each of these measures.

The overall positive ratings for self-efficacy in this study are consistent with the observation by Pajares (2003) in his review of the literature that students in the earliest grades believe they can write. The present study provides the first evidence that children as early as Grade 2 are confident about their capability to self-regulate writing, at least for the types of skills assessed by the measure used in this study. Further research is needed to replicate this positive sense of efficacy and to expand its exploration. For instance, it would be helpful to know how beginning writers’ efficacy for writing self-regulation compares to their efficacy for generating and organizing ideas when writing; efficacy for applying foundational writing skills such as handwriting, spelling, grammar, and sentence construction; and efficacy for successfully completing writing tasks that vary in difficulties (e.g., writing a sentence, writing a paragraph, writing a story). It would also be fruitful to examine if providing students with a referent for judging their self-efficacy for writing self-regulation would influence judgments. For example, students could be asked to judge their capabilities in comparison to their classmates (see Graham et al., 1993). This may change young students’ sense of efficacy for writing self-regulation because it provides a more concrete reference point for considering this capability.

The overall positive ratings for attitude toward writing in the current study are also consistent with the conclusions drawn by Ekholm et al. (2018) that beginning writers are positive about writing. Additional research is needed to replicate this finding, as well as our finding concerning self-efficacy for writing self-regulation, with students from different countries and cultures. Motivational beliefs such as these are not culturally or contextually neutral (see Klassen et al., 2009; Graham et al., 2022). Further, our measure of writing attitudes was directly tied to compositions that students wrote. It would be interesting to determine if similar outcomes are obtained with beginning writers when this is not the case or when students are asked to evaluate what they wrote before making an attitudinal judgment.

As predicted, girls were more confident than boys about their writing self-regulation capabilities. They also expressed a more positive attitude toward writing than boys. These findings are consistent with outcomes reported in previous investigations (Pajares, 2003; Ekholm et al., 2018). Additional research is needed to determine why such gender differences occur. It is possible that the observed differences in writing motivational beliefs between girls and boys was not a function of gender *per se*, but a consequence of gender stereotypical beliefs. For instance, Pajares and Valiante (2001) found that gender differences in middle school students’ writing self-efficacy were no longer evident when their gender orientation beliefs were considered. It is possible that children believe that writing is more of a feminine-domain, fostering the belief that girls are more competent writers than boys. We think that is especially important for teachers and parents to address such stereotypes. One way of doing this is for adults to consistently express the opinion that writing is the domain of both boys and girls and both groups of children can each be effective and successful writers.

Our prediction concerning the relationship between students’ language status (Norwegian as first language, Norwegian and another language both as first language, or Norwegian as second language) and self-efficacy for writing self-regulation and attitude toward writing was only partially supported. Language status was not statistically related to self-efficacy for writing self-regulation, but students who had both Norwegian and another language as a first language as well as students for whom Norwegian was a second language had a more positive attitude about writing than student with just Norwegian as a first language. It is possible that students who were native speakers of Norwegian and another language had more positive attitudes because learning two languages boosted their cognitive and/or language capabilities (e.g., Bialystok, 2001). Since writing is a cognitive activity that relies on language skills to express ideas and thoughts, this may have enhanced students’ writing, resulting in a more positive valence toward writing. Unfortunately, this explanation is at odds with our findings that students learning Norwegian as a second language had higher a more positive attitude toward writing than native speaking Norwegians. It is possible that students learning Norwegian as a second language may have interpreted the items on the attitude scale differently than the other two groups of children. It is also possible that students who are still learning Norwegian are more positive than native speakers about the opportunity to write in this new language. In any event, assuming our findings concerning language status, writing attitudes, and self-efficacy are replicated, additional research will be needed to untangle these relationships.

### Efficacy, attitude, and writing quality

As predicted, both self-efficacy for writing self-regulation and attitude toward writing each made a statistically significant and unique contribution to predicting the quality of Grade 2 students’ writing after controlling for the other writing belief, age, handwriting fluency, and school-level factors of school size, national test scores, proportion of certified teachers, school hours per student, and number of students per special education teacher. These findings are generally consistent with outcomes in previous research conducted mostly with older students (Pajares, 2003; Ekholm et al., 2018). Our findings replicated the work of Graham et al. (2007, 2012) showing that attitudes toward writing can predict the writing performance of beginning writers but extends previous work involving writing self-efficacy by demonstrating that efficacy for writing self-regulation predicts the quality of beginning writers’ text.

Any claims derived from these findings about the predictive value of self-efficacy for writing self-regulation and attitudes toward writing must be mitigated by fact that collectively these two writing motivational beliefs accounted for only 2% of the variability in the quality of students’ text once variability associated with handwriting fluency, age, gender, language status, and the five school-related variables were controlled. This raises questions about the possible theorized effects on writing of these two writing motivational constructs for beginning writers (Hayes, 1996; Zimmerman and Risemberg, 1997; Graham, 2018). Further, the finding that self-efficacy for writing self-regulation only accounted for a unique 1% of the variance in writing quality was consistent with the claim by Berninger and Amtmann (2003) that self-regulation effects take time to be realized. It is possible that in second grade the hypothesized effects of writing efficacy and attitudes on students’ writing are weaker than anticipated or that their effects are indirectly realized through their interaction and association with other individual and contextual variables. Assuming that our results are replicated, models can be derived and tested to more precisely determine the direct and indirect effects of our two writing motivational measures.

It is also important to realize that our study only assessed one aspect of self-efficacy (self-regulation) and our measure of writing attitude included items that assessed the affective and cognitive aspects of attitude, but not motivational ones (see Ekholm et al., 2018). Future research needs to expand the attributes of writing efficacy and attitudes assessed with children this young. It is possible the inclusion of other aspects of these two writing motivational beliefs will strengthen their predictive value. Further, the findings from this investigation may underestimate the predictive value of self-efficacy for writing self-regulation and attitude toward writing. The means for both measures were relatively high and the standard deviations large enough that ceiling effects were possible. Ceiling effects can attenuate relationships between predictors and outcome measures, resulting in smaller correlation (Nunnally and Bernstein, 1994). Thus, future research in this area with beginning writers will need to address this issue.

While gender, language status, handwriting fluency, and national test scores of participating students’ schools were control variables, each made a unique and statistically significant contribution to predicting the quality of students’ writing. These outcomes are consistent with prior research demonstrating that girls are better writers than boys (Reilly et al., 2018), writing outcomes differ by language status (Camping et al., 2020), handwriting fluency is related to quality of students’ writing (Graham et al., 1997; Kent and Wanzek, 2016; Skar et al., 2022), and school-level factors predict students’ writing performance (Walberg and Ethington, 1991).

### Limitations and implications

When interpreting the results of this investigation, it is important to keep three limitations in mind. One, while the study sample was large and representative of Grade 2 students in Norway, the findings do not necessarily generalize to countries with different social and cultural backgrounds. Likewise, such effects may vary depending upon the curricula and instructional approach to writing that is emphasized. We suspect that research conducted in countries with similar cultural, social, institutional, historical, and political backgrounds to Norway would be more likely to produce similar findings to ours than countries that differ significantly on one or more of these factors (Graham, 2018). Students exposed to similar instructional or curricular materials would also be more likely to yield similar patterns of relationships than writing programs that differ considerably. However, additional research is needed to substantiate these predictions.

Two, while students wrote two different texts as part of the study, these texts did not represent the full range of writing that young Norwegian students commonly complete at school (see Graham et al., 2021). Thus, the relations obtained in this study between text quality, efficacy for writing self-regulation, and attitude toward writing may differ when different kinds of writing are investigated. Three, we did not assess all aspects of writing efficacy and attitudes, and outcomes could differ depending on what is tested. Four, we did not administer a test of language proficiency in this investigation. This may have provided a better measure of language status versus whether students were classified as L1, L2, or bilingual. Finally, future investigations could include more measures, such as teachers’ educational background to add even more precision in the model.

Despite these limitations, the current study demonstrated that the young beginning writers in this study were positive about their efficacy for writing self-regulation and had positive attitudes about what they wrote. Because some students’ positive beliefs about writing can decline over time (Pajares, 2003; Graham, 2006), we encourage teachers to nourish students’ writing confidence and views about writing as they progress through the grades. Moreover, girls in this study created texts that were judged to be of higher quality than text produced by boys. They also viewed writing more positively and were more efficacious about their capabilities to self-regulate writing. Like Pajares (2003), we think these differences were a result of gender stereotypic beliefs about writing and not gender *per se*. A challenge for teachers and parents, therefore, is to change children’s view of writing so that it is perceived as valuable, relevant, and pertinent for both boys and girls.

The self-efficacy for writing self-regulation and attitude toward writing measure used in this study collectively accounted for only 2% of the variance in the quality of students’ writing. It is possible that this was the case because the young children in this study had limited opportunities to form such judgements about such beliefs, attenuating their possible effects on students’ writing. Teachers can potentially strengthen these linkages by increasing how much students’ write; providing them with positive writing experiences, asking students to identify how the processes, strategies, and skills they apply strengthen their writing; and providing such feedback to students themselves.

Finally, while the findings of this study are descriptive and correlational, and great care must be taken in drawing educational implications from such data, we offer the following observations for educational practice. First, because attitude toward writing and self-efficacy for self-regulation each uniquely predicted the quality of young students’ writing, teachers want to keep both of these motivational beliefs in mind when teaching writing. This includes putting into place procedures known to promote a positive sense of efficacy as well as attitude toward writing. For instance, teachers can potentially promote efficacy for self-regulation by engaging students in tasks where they successfully regulate the writing process (i.e., mastery experiences), observe other students or the teacher use writing self-regulation procedures successfully (i.e., vicarious experiences), and by telling students they are capable of regulating the writing process or providing them with feedback when they do so (i.e., persuasion). As Bandura (2006) noted, each of these sources of information can enhance efficacy. In terms of attitude toward writing, teachers can potentially promote a positive point of view by providing students with choice when selecting writing topics, having students work together when writing and supporting students as they write to ensure success, and teaching students needed and important writing skills and strategies (Ekholm et al., 2018). Second, the findings from this study suggest that primary grade teachers need to monitor the attitudes and efficacy of the all students in their class. For example, boys are likely to view writing more negatively than girls and believe they are less efficacious than girls with regards to their capabilities to regulate the writing process. This may well be due to stereotypical beliefs that girls are better writers than girls. Teachers and parents need to actively promote a different view—both boys and girls are capable writers. This belief needs to be stated frequently and reinforced. Likewise, based on the findings from this investigation, it is important to monitor the writing attitudes of young students whose language status differ and apply the types of instructional procedures identified above that promote more positive attitudes toward writing.

## Author’s note

This study was registered with the Norwegian center for research data (Identifier 848410).

## Data availability statement

The raw data supporting the conclusions of this article will be made available by the authors.

## Ethics statement

The studies involving humans were approved by the Norwegian Center for Research Data (Identifier 848410). The studies were conducted in accordance with the local legislation and institutional requirements. Written informed consent for participation in this study was provided by the participants’ legal guardians/next of kin.

## Author contributions

GS: Conceptualization, Formal analysis, Funding acquisition, Investigation, Methodology, Writing – original draft. SG: Conceptualization, Formal analysis, Investigation, Methodology, Writing – original draft. AH: Conceptualization, Formal analysis, Investigation, Methodology, Writing – original draft.

## Footnotes

1 Data for gender, and language comparisons stem from Information System of the primary and lower secondary schools in Norway [i.e., Grunnskolens informasjonssystem (GSI); https://gsi.udir.no/].

2 There is no official data on proportions of schools in Norway located in rural areas.

3 Unfortunately, writing is not tested in Norway until 10th grade as (and then as a part of the language arts exam), and the general academic proficiency tests in reading, mathematics and English are taken by students in 5th grade.

4 The classical test theory reliability between raters, when considering the anchor texts (i.e., the same 50 texts across all 24 raters) was ICC = 0.98 [0.97–0.99] for audience awareness, ICC = 0.98 [0.97–0.99] for organization, ICC = 0.99 [0.99–0.99] for content relevance, ICC = 0.98 [0.96–0.98] for vocabulary, ICC = 0.98 [0.97–0.99] for sentence construction, ICC = 0.96 [0.95–0.98] for spelling, ICC = 0.98 [0.97–0.99] for legibility and ICC = 0.99 [0.98–0.99] for punctuation.

5 This was an intervention project targeting writing development. The intervention failed to produce differences in text quality, handwriting fluency and attitude toward writing between students in the control group and in the intervention group (Skar et al., 2023). Because of this we have opted to not to include any subgroup (i.e., control group, intervention group) analyses in this investigation.
