# Supplementary material for: Efficacy for writing self-regulation, attitude toward writing, and quality of second grade students’ writing
Source: Front Psychol. 2023 Oct 17;14:1265785. doi: 10.3389/fpsyg.2023.1265785 (PMC10616524; doi:10.3389/fpsyg.2023.1265785)
Supplement: Supplementary file 1 [file Data_Sheet_1.docx]

**APPENDIX A – RATING SCALES**

|  | **Level 1** | **Level 2** | **Level 3** | **Level 4** | **Level 5** |
| --- | --- | --- | --- | --- | --- |
| **Audience Awareness** | To understand the text, a conversation with the writer is required. | The text contains words/characters/drawings that make sense in interaction with each other. | The text contains elements that indicate that the text addresses a reader. | The text addresses the reader in the assignment in a fairly relevant manner and takes into account to some extent the reader’s need for knowledge of participants/characters, circumstances, and events. | The text addresses the reader in the assignment in a generally relevant manner and takes into account the reader’s need for knowledge of participants/characters, circumstances, and events.  The text may contain traces of the student’s voice with reflective or evaluating utterances. |
| **Organization of content** | The text consists of individual letters/words/characters/drawings. | The text may indicate a structure, such as in the form of a list with a marked thematic headline or letter structure.  The additive connector “and” may appear. | The text has a global structure with elements arranged in a logical order. In some cases, the introduction or ending may not be explicit.  The text contains primarily additive and temporal connectors (e.g. “and,” “so.” | The text has a global structure with some elaborated elements arranged in a logical order. In some cases, the introduction or ending may not be explicit.  The text may show examples of comparisons, classifications, chronology.  The text includes different connectors (e.g., “but,” “also,” “because”). | The text has a complete global structure with several elaborated elements arranged in a logical or otherwise appropriate order.  The text contains connectors that are used suitably and purposefully. |
| **Content Relevance** | The part of the verbal text that is a relevant answer to the task corresponds to a sentence or less. | The part of the verbal text that is a relevant answer to the task corresponds to approx. two to three sentences. | The part of the verbal text that is a relevant answer to the task corresponds to approx. half an A4 page (25–49 words). | The part of the verbal text that is a relevant answer to the task corresponds to approx. an A4 page (50–74 words). | The part of the verbal text that is a relevant answer to the task corresponds to approx. one and a half A4 pages or more (75+ words). |
| **Vocabulary** | The text consists of individual letters/words/characters/drawings. | The text contains some few (different) words. | The text contains several different words (a lot of them theme-related). | The text contains a repertoire of words and expressions (a lot of them theme-related). | The text contains a repertoire of words and expressions (a lot of them theme-related).  In some cases, there is use of specialized and abstract words and/or creative forms of expression. |
| **Language use (Sentence construction)** | The text consists of individual letters/words/characters/drawings. | There may be complete sentences. | The sentences show little variation in structure (in texts where variation is relevant). | Parts of the text shows appropriate variation in sentence structure. | The text has for the most part appropriate syntactic variation, and it has some developed phrases and/or subordinate clauses. |
| **Punctuation** | The text has no punctuation. | Some punctuation can occur and/or there is exploratory use of punctuation. | Occurrences of functional use of punctuation (especially dot). | Mostly correct use of periods. Occurrences of functional use of question marks and/or exclamation marks (in texts where relevant). | Functional use of various forms of punctuations. The use of a comma may occur. |
| **Spelling** | There may be letters in the text and/or there may be scribbles (imitating writing). | The text contains letter combinations and single words. | The words are spelled phonetically, and some high-frequency words related to primary school students’ sphere are written correctly. | There are examples of non-phonetic words that are correctly written. There may be examples of overgeneralization (for example, silent “h” first in words starting with “v” - hvært). | There are a number of examples of non-phonetic words written correctly. |
| **Handwriting (legibility)** | Letters may be difficult to decipher (if any). | The text contains letters that are not crafted in a conventional manner. | The letters are mainly crafted in a conventional manner. | The letters are crafted in a conventional manner.  Instances of conventional use of the “handwriting house.”*  Occurrences of alternating use of upper- and lower-case letters. | The letters are drafted in a conventional and legible manner.  For the most part, there is conventional use of the “handwriting house.”  Usually follows conventions for use of upper- and lower- case letters. |

*The “handwriting house” denotes the relative position of letters. A lower-case “g” and a lower-case “h” will—if correctly written—be placed in the “ground floor” and “basement” (g) and in the “ground floor” and “addict” (h).
